# Impact of the COVID-19 pandemic on patients with rheumatic and musculoskeletal disease in the UK and Europe: the REUMAVID study (Phase 1)

**DOI:** 10.1093/rap/rkab098

**Published:** 2021-12-04

**Authors:** Stephanie Rose Harrison, Marco Garrido-Cumbrera, Victoria Navarro-Compán, José Correa-Fernández, Dale Webb, Laura Christen, Helena Marzo-Ortega

**Affiliations:** 1 National Institute of Health Research (NIHR) Leeds Biomedical Research Centre, Leeds Teaching Hospitals Trust, Leeds, UK; 2 Leeds Institute of Rheumatic and Musculoskeletal Medicine (LIRMM), University of Leeds, Leeds, UK; 3 Health and Territory Research, Universidad de Sevilla, Seville, Spain; 4 Axial Spondyloarthritis International Federation (ASIF), London, UK; 5 Hospital Universitario La Paz, IdiPAZ, Madrid, Spain; 6 National Ankylosing Spondylitis Society (NASS), London, UK; 7 Novartis Pharma AG

**Keywords:** Rheumatic and musculoskeletal diseases, COVID-19, Pandemic, Wellbeing, Mental health, United Kingdom, Europe

## Abstract

**Objectives:**

To compare the impact of the first wave of the COVID-19 pandemic and lockdown measures on patients with rheumatic and musculoskeletal diseases (RMDs) in the UK and other European countries (OEC).

**Methods:**

REUMAVID was an online cross-sectional survey of seven European countries. Data collected included: demographics, lifestyle, employment, access to healthcare services, disease-specific characteristics, WHO-5 Well-Being Index, Hospital Anxiety and Depression Scale (HADS), Visual Analogue Scale (VAS) disease activity, and the Perceived Acceptable Symptom Scale.

**Results:**

1,800 responses were received between April and July 2020 [UK, n = 558(31.0%); OEC, n = 1,242(69.0%)]. UK patients were more likely to be older (UK 58.5years±13.4; OEC 50.0years±12.2), university educated [UK n = 302(54.1%); OEC n = 572(46.1%), quit smoking [UK n = 92(59.4%); OEC n = 65(16.2%)] and continue exercise [UK, n = 216(49.2%); OEC, n = 228(33.1%)], although conversely alcohol consumption increased [UK n = 99(36.3%); OEC n = 98(12.1%)]. UK patients felt informed about COVID-19 (UK 72.7%, OEC 57.4%) and kept their planned rheumatology [UK n = 87(51.2%); OEC n = 213(38.6%)] and/or GP appointments [UK n = 87(76.3%); OEC n = 310(53.9%)]. Almost half of patients with RMDs reported a decline in health and wellbeing, though this was less common in UK patients [UK n = 214(38.4%), OEC n = 618(50.2%)] who reported better perceived acceptable symptom scale, VAS pain and HADS scores, but worse WHO-5 scores.

**Conclusions:**

UK RMD patients performed better in the physical and mental health domains tested possibly due to a less restrictive lockdown and improved healthcare access. These findings have implications for healthcare services globally in planning patient care after the COVID-19 pandemic.

**Key messages**

- The COVID-19 pandemic negatively affected the physical and mental health of European RMD patients.
- Compared with other European counterparts, UK patients performed better in nearly all health domains tested.
- The insights from this survey could help shape the care of RMD patients post-pandemic.

## Introduction

The COVID-19 pandemic has, and continues to, put an unprecedented strain on healthcare systems globally. The speed at which both community and hospital healthcare services reorganised to accommodate the influx in COVID-19 patients was remarkable, however non-COVID-19 acute and elective services suffered significantly as a result (1,2). Patients with rheumatic and musculoskeletal diseases (RMDs) are potentially more likely to be adversely affected due to a combination of factors, including reduced detection of flare or adverse treatment effects, lack of continuity of care and access to specialist support and the requirement for stricter social distancing and shielding practices for those taking immunosuppressive medications (3–6). In addition patients with RMDs are known to have associated morbidities and poorer baseline physical and mental wellbeing when compared with otherwise healthy individuals (7–9).

Strategies to manage the impact of the pandemic locally, regionally, nationally, and internationally have varied vastly, reflecting both rapidly evolving research and understanding of the SARS-CoV-2 virus, and the inherent differences between populations, healthcare systems and the wider socio-economic context (10). Patients and clinicians alike faced an insurmountable task in interpreting conflicting information from the media, healthcare organisations, charities and governments (11) with individual nations adopting different containment measures; for example, while outdoor activities and exercise were permitted in the UK in Spain and Italy people were largely confined to indoors (10,12). Crucially, whilst stricter lockdown measures may provide more significant reassurance of protection from the virus, the detrimental effects on general health and wellbeing are also potentially greater. A better understanding of the impact of the pandemic on patients with RMDs is now needed to help rheumatologists and healthcare systems address its short- and long-term health effects (13).

The REUMAVID study aimed to assess the impact of the COVID-19 pandemic in people with rheumatic and musculoskeletal disorders across European countries. In the present study we compared the impact of the first wave of the COVID-19 pandemic and associated lockdown measures on rheumatology patients in the United Kingdom (UK) with RMD patients in other European countries (OEC).

## Methods

REUMAVID is a multidisciplinary international consortium assembled at the start of the pandemic with the aim of assessing its impact on the physical/ mental health and overall wellbeing of patients with RMDs from participating European countries (Cyprus, France, Greece, Italy, Portugal, Spain and the

UK), as described in detail previously (14). Patients were recruited through national RMD patient groups who disseminated the survey link via their organisational website. All participants were aged 18 or above, residing in a REUMAVID-participating country, who had or were expected to have an appointment with their rheumatologist within 12 months of study entry. Prior to completing the survey all patients provided informed consent electronically via the online survey platform. REUMAVID was first approved by the ethical committee of University Hospital La Paz under the code PI-4121 and was subsequently approved in all other participating countries as legally required.

Patients first self-reported their diagnosis from the following list; ankylosing spondylitis/ axial spondyloarthritis, fibromyalgia, gout, juvenile idiopathic arthritis, myositis (polymyositis or dermatomyositis), osteoarthritis, osteoporosis, peripheral spondyloarthritis, polymyalgia rheumatica, psoriatic arthritis, rheumatoid arthritis, synovitis, acne pustulosis hyperostosis and osteitis (SAPHO), Sjögrens, systemic lupus erythematosus, systemic sclerosis (or scleroderma), vasculitis or arteritis.

They then completed a 120-question survey covering multiple domains of physical and mental health and wellbeing including demographics, lifestyle habits and weight, provision of information on COVID-19, measures of disease activity and mental/ physical health, and four validated scales to measure wellbeing: (i) the WHO five well-being index (WHO-5) which measures overall wellbeing (15); (ii) the Hospital Anxiety and Depression Scale (HADS) to measure anxiety/depression levels (16,17); (iii) Visual Analogue Scale (VAS) for disease activity and (iv) the Patient Acceptable Symptom Scale (18)..

Survey data was analysed using SPSS v26.0. For continuous parametric variables, the mean and standard deviation are reported. Statistical significance between groups (the UK and OEC) were determined using t-test for individual variables. Categorical outcome variables are report as a frequency/ percentages and statistically significant difference between the UK and OEC were determined using $ChiX_2$ test. Sample size is reported for each individual variable to highlight any areas of missing data.

**Results**

Overall, 2,731 patients with RMDs participated in the first phase of the study. Due to the patients completing <70% of the survey, data for 931 patients had to be discarded , leaving 1,800 patients for data anaysis, of which 558 respondents (31%) were recruited by the National Ankylosing Spondylitis Society, the National Rheumatoid Arthritis Society and Arthritis Action in the UK.

Demographics

Compared with OEC, UK participants were older (UK 58.5 years ± 13.4; OEC 50.0 years ±12.2, p<0.001). Overall, the vast majority of respondents were female both in the UK and the OEC [UK 78.7%, (439), OEC 81.0% (1,003), p=0.262] and identified themselves as being married or in a relationship [UK 70.8% (395), OEC 69.1%, (858), p=0.008] with similar proportions of respondents from both the UK and OEC being members of patient organisations [UK 42.4% (236), OEC 41.2% (512), p=0.627] [Table 1]. Contrastingly, UK participants were more likely to have obtained a university qualification [UK 54.1% (302), OEC 46.1%, (572), p<0.001] and to be retired [UK 35.5% (198), OEC 16.5% (205), p<0.001] [Table 1]. Interestingly, amongst working participants teleworking was commoner in the UK [UK 58.1% (144), OEC 32.9% (215), p<0.001] [Table 1].

Weight and lifestyle

Over half of study participants were overweight or obese [Body Mass Index (BMI) >25; UK 57.4%, (190), OEC 54.1% (640)] with rates of obesity (BMI >30) being higher in the UK population [UK 31.4% (104), OEC 23.0% (272), p=0.011] [Table 1]. In addition, 39.2% (219) of UK participants and 41.6% (517) in the OEC reported gaining weight during the first wave period (p=0.561). Almost one third of participants reported smoking (31%, UK=155, OEC=401), although in the UK, 59.4% (92) of participants said they had quit smoking, compared to just 16.2% (65) in OEC (p<0.001) [Table 1]. The converse was true for alcohol consumption with 60.3% of all respondents reporting alcohol consumption, with UK participants admitting to drinking more [UK 36.3% (99), OEC 12.1% (98), p<0.001] or the same amount [UK 50.2% (137), OEC 32.9% (267), p<0.001] compared with their European counterparts whereas nearly half of respondents in the OEC did not drink at all [UK 0.4% (1), OEC 42.7% (347), p<0.001] [Table 1]. UK participants were more likely to have continued physical activity during the pandemic [UK 49.2% (216), OEC 33.1% (228), p<0.001], to visit a natural environment [UK 51.9% (288), OEC 31.5% (275), p<0.001], and/or go for daily walks outside [UK 38.4% (214), OEC 22.2% (276), p<0.001]. They were also more likely to do grocery shopping online [42.4% (65), OEC 17.8% (78), p<0.001] whilst in OEC, patients would report someone from their household going to the shop in person (p<0.001) [Table 1].

Access to rheumatology services

More than half of rheumatology patients in the UK were able to keep scheduled rheumatology appointments during the pandemic which was more than in the OEC (UK 51.2% vs. 38.6%, p=0.004) [Table 2]. They were also more likely to be able to discuss the effects of treatment on COVID-19 with either their rheumatologist (81.0% vs.57.6%, p<0.001) or general practitioner (76.3% vs. 53.9%, p<0.001). By contrast, around half of patients reported not being able to continue their psychological/ psychiatric therapies either online or via telephone, with no significant difference in the UK compared with OEC (45.7% vs.52.2%, p=0.459) [Table 2].

Access to information on COVID-19

Overall, 35.4% in the UK reported receiving no information on how COVID-19 may affect their RMD, compared with 51.6% in the OEC [Figure 1, p<0.001]. Rheumatologists were the main source of information for patients on how COVID-19 may affect their RMD, with twice as many UK patients (37.9%) patients reporting receiving such information versus those in the OEC (19.0%) [Figure 1]. The next commonest source for this information were patient organisations (UK 33.3%, OEC 25.9%), followed by general practitioners (UK 19.2%, OEC 13.4%), and the national rheumatology societies (UK 9.4%, OEC 12.2%) [Figure 1].

Interestingly, whilst patient organisations played an important role in providing information for patients in all countries, the focus of the information provided differed. For instance, in the UK, patient organisations provided more COVID-19 related information (UK 72.7%, OEC 57.4%) whilst in the OEC, patient groups provided more disease-related information (UK 30.3%, OEC 59.1%, p=0.017) [Figure 2]. Less information was provided overall on how to access their rheumatologist, home delivery of treatments and emotional/psychological support in both the UK and OEC [Figure 2].

Disease-specific characteristics and measures of overall health/ wellbeing

In the UK, 54.2% (302) of patients reported good or very good self-perceived health, compared with 27.8% (342) in OEC (p<0.001), and UK patients were less likely to report a deterioration in their health, with 38.4% (214) in the UK reporting worsening of their health compared with 50.2% (618) in the OEC (p<0.010) [Table 3]. More than two thirds of patients in the UK and OEC reported increased disease activity, which was slightly less in the UK cohort [UK 67.6% (374), OEC 76.5% (920), p<0.001] [Table 3]. Levels of anxiety and depression were also worse in the OEC [HADS anxiety, UK 43.6% (241), OEC 63.5% (772), p<0.001; HADS depression UK 33.6% (186), OEC 51.4% (625) (p<0.001)] although poorer overall wellbeing was reported by UK patients [WHO-5 ≤50; UK 52.5% (292), OEC 47.3% (578), p=0.043] [Table 3].

## Discussion

As the focus shifts from immediate treatment of SARS-CoV-2 infected patients and towards mitigating the effects of the pandemic on patients with chronic health conditions, there is a significant need to better understand indirect effects of the ongoing COVID-19 pandemic on the overall wellbeing and disease activity of patients with RMDs. This survey compared and contrasted the experience of UK patients with that of OEC during the first wave of the COVID-19 pandemic. We felt that this analysis was of interest as political decisions led to different approaches in containment and lockdown measures throughout Europe. British respondents were generally older, with higher educational attainment and more likely to be retired which is similar to previous surveys (19,20). As expected from the less stringent containment measures in the UK, more British patients continued exercise, often outdoors or on a natural environment. They also reported quitting smoking, all of which have positive impacts on physical and mental health, although conversely many UK respondents increased their alcohol consumption. Teleworking and online/telephone grocery shopping were also commoner in the UK. British patients were more likely to keep their appointment with their rheumatologist or GP, received more information on COVID-19 and had higher levels of satisfaction with the quality of that information. Self-reported health status, anxiety and depression levels were better in UK patients, although overall levels of wellbeing were comparatively worse, possibly due to the self-reported nature of the instrument used (21).

Despite the comparatively better outcomes for British patients there was still a significant burden associated with lockdown measures. One reason for this was the significant challenges all healthcare organisations faced in assimilating and disseminating high quality guidelines in a rapidly changing research environment with no clear standard of best practice. At the start of the pandemic, EULAR published principals for the care of patients with RMDs, however there were significant ramifications to their recommendations as well as many obstacles to their implementation, with lockdown, redeployment of healthcare staff and strict social distancing (22). As a result, less than half of patients kept their rheumatology appointment (UK 51.2%, OEC 38.6%, p=0.004), the impact of which is reflected in the self-reported disease-specific characteristics seen in this survey.

Nevertheless, British patients still fared better overall, which may be partly due to less stringent government restrictions. Researchers at University of Oxford developed a scoring system to quantitatively compare the stringency of lockdown measures between countries (10). Using this resource, between January and June 2020, the UK had the least strict measures compared with the OEC. Since adherence to lockdown rules in the first wave was high, especially amongst patients with RMDs who were considered vulnerable/ extremely vulnerable (23,24), it seems reasonable to assume that the stringency of lockdown measures would impact on overall health and wellbeing.

One notable difference between lockdown measures was that British patients were more likely to continue to exercise outdoors. Exercise and outdoor activity reduce anxiety and depression, improve overall wellbeing (25,26), maintain mobility and reduce pain in patients with RMDs (5). British patients also felt they received better-quality RMD-specific guidance on COVID-19, which likely provided some reassurance at a time of great uncertainty. In addition, more UK patients reported already belonging to patient organisations before the pandemic and it is possible that having these support networks already established, helped British patients to feel more connected to others in their position, and facilitated rapid access information, making them feel empowered. Furthermore, UK patients appeared to use more digital technologies; for example in grocery shopping online and teleworking which may have provided a routine and some semblance of normality, contributing to improved their resilience during the first wave.

This study is the first to report the impact of the first wave of the COVID-19 pandemic on the physical/ mental health and overall wellbeing of patients from seven participating European countries, providing valuable insight into the impact associated to the different containment measures initiated in each country. These results can inform the future planning and delivery of services for patients with RMDs in Europe. There are some limitations, however, similar to other survey studies, with an overrepresentation of females, those who are retired and those with higher levels of educational attainment (27). Nevertheless, the sex distribution may be related to the distribution of rheumatic disease in the survey, although approximately one third of respondents had axial spondyloarthritis, which is more frequently diagnosed in males. Those with higher educational attainment are also more likely to engage with patient support groups, self-help measures and to be able to easily access and understand the rapidly evolving information on the pandemic. Conversely, those who work may have been deterred from participation in the survey due to the length of time it would take to complete 120 questions, and those from lower socio-economic background and/or ethnic minority groups may not have access to adequate digital facilities or be able to access the survey in their native language (28). Thus, the relative impact of the pandemic on those of working age, males and those from deprived background or ethnic minority groups is unclear. In addition, the distribution of RMDs in this survey does not reflect the prevalence of RMDs in real-world practice, with overrepresentation of axial spondyloarthritis and rheumatoid arthritis, reflecting the instrumental role played by the patient societies collaborating in the REUMAVID study.

Furthermore, the patient survey does not allow us to detect inter-regional and local differences in the patient experience, which is key information for care providers who need to effectively utilise already stretched local resources in the aftermath of the first wave of the pandemic. This is even truer of the second wave of the pandemic in the UK, where inter-regional lockdown practices differed significantly compared with the first wave. Future surveys should therefore seek to target under-represented groups and provide local and regional data. This survey is cross sectional, and as alluded to previously experiences may well differ between different phases of the pandemic, even within the same country. Similarly, our survey does not include all European countries, and the patient experiences there, or indeed in non-European nations, could differ quite substantially. Finally, there is no control group of patients completing the survey prior to the pandemic, although in our survey patients, when asked, did report that their health had worsened relative to the pre-pandemic state.

In conclusion, these data suggest that UK patients with RMDs performed better than OEC in the physical and mental health domains tested. . UK respondents reported less smoking and greater levels of physical activity, although they consumed more alcohol, showed greater adaptation to digital platforms with increased use of online shopping and teleworking.. These differences may be due to a multitude of factors, both known and unknown. Nevertheless, the REUMAVID survey does highlight some factors that could have played a role in the observed differences including the fact that UK patients were living under comparatively less stringent lockdown measures with better access to outdoors spaces, healthcare professionals, and advice on COVID-19. Irrespective of the reasons behind the differences, the REUMAVID survey highlights the clear negative effects of the first wave on their mental health all patients with RMDs, even if UK respondents reported comparatively less anxiety and depression. These findings have broad implications for healthcare services globally in planning patient care in the aftermath of the pandemic.

**Acknowledgements**

We would like to thank all of the patients who completed the survey, as well as all of the patient organisations and patient representatives who participated in the REUMAVID study including: Souzi Makri from the Cyprus League Against Rheumatism (CYPLAR) from Cyprus, the Association Française de Lutte Anti-Rhumatismale (AFLAR) from France, Katy Antonopoulou from the Hellenic League Against Rheumatism (ELEANA) from Greece, Serena Mingolla from the Associazione Nazionale Persone con Malattie Reumatologiche e Rare (APMAAR) from Italy, Elsa Frazão Mateus from the Portuguese League Against Rheumatic Diseases (LCPDR) from Portugal, Pedro Plazuelo Ramos from the Spanish Federation of Spondyloarthritis Associations (CEADE), the Spanish Patients' Forum, UNiMiD, Spanish Rheumatology League (LIRE), Andalusian Rheumatology League (LIRA), Catalonia Rheumatology League and Galician Rheumatology League from Spain, and Jim Hamilton from the National Axial Spondyloarthritis Society (NASS), Clare Jacklin and Ailsa Bosworth from the National Rheumatoid Arthritis (NRAS), Shantel Irwin from Arthritis Action from the UK. We would further like to thank Patricia Pertel, Maria-Magdalena Balp, and Marina Milutinovic of Novartis Pharma AG for their contributions to the study design and execution.

HM-O is supported by the National Institute for Health Research (NIHR) Leeds Biomedical Research Centre (LBRC). The views expressed are those of the authors and not necessarily those of the (UK) National Health Service (NHS), the NIHR, or the (UK) Department of Health.

**Funding statement:** The REUMAVID study is supported by Novartis Pharma AG.

**Disclosure statement:** HM-O reports grant/research support from: Janssen and Novartis, consultant for: AbbVie, Celgene, Janssen, Lilly, Novartis, Pfizer and UCB, speakers' bureau: AbbVie, Biogen, Celgene, Janssen, Lilly, Novartis, Pfizer, Takeda and UCB. HM-O is supported by the National Institute for Health Research (NIHR) Leeds Biomedical Research Centre. LC is an employee of Novartis Pharma AG. JC has received grant funding from Abbvie, Amgen, Biogen, Eli Lilly, Gilead, Janssen, Pfizer, Roche, Sanofi and UCB. DW has received grant funding from AbbVie, Biogen, Janssen, Lilly, Novartis and UCB. SH was sponsored to attend a medical conference by UCB and financial reimbursement for speaking at an educational event organised by UCB. VN-C reports honoraria/research support from: Abbvie, BMS, Janssen, Lilly, MSD, Novartis, Pfizer, Roche and UCB. The remaining author has declared no conflicts of interest.

**Data availability statement:** Please direct any requests for access to raw data to the corresponding author.

**Table 1. Socio-demographic, working life, anthropometric characteristics, lifestyle habits and outdoors contact during the COVID-19 pandemic**.

| | **Mean ± SD; n (%)** | | **p-value** |
|---|---|---|---|
| | **UK** 558 (31.0%) | **OEC** 1,242 (69.0%) | |
| **Age** (years) | 58.5 ± 13.4 | 50.0 ± 12.2 | **<0.001*** |
| **Gender** (female), n: 1,797 | 439 (78.7) | 1,003 (81.0) | 0.262 |
| **Marital status** | | | |
| Single | 81 (14.5) | 206 (16.6) | **0.008*** |
| Married / in relationship | 395 (70.8) | 858 (69.1) | |
| Separated/divorced | 51 (9.1) | 144 (11.6) | |
| Widower / widow | 31 (5.6) | 34 (2.7) | |
| **Educational level** | | | |
| No schooling completed | 13 (2.3) | 7 (0.6) | **<0.001*** |
| Primary school | 0 (0.0) | 72 (5.8) | |
| Secondary school | 94 (16.8) | 213 (17.1) | |
| Vocational qualification | 149 (26.7) | 378 (30.4) | |
| University | 302 (54.1) | 572 (46.1) | |
| **Employment status** | | | |
| Retired | 198 (35.5) | 205 (16.5) | **<0.001*** |
| **Teleworking,** N: 901 | 144 (58.1) | 215 (32.9) | **<0.001*** |
| **Patient organisation** (member) n: 1,798 | 236 (42.4) | 512 (41.2) | 0.627 |
| **Body Mass Index,** n: 1,513 | | | |
| Underweight (< 18.5) | 14 (4.2) | 41 (3.5) | **0.011*** |
| Normal weight (18.5–24.9) | 127 (38.4) | 501 (42.4) | |
| Overweight (25–29.9) | 86 (26.0) | 368 (31.1) | |
| Obesity (> 30) | 104 (31.4) | 272 (23.0) | |
| **Gaining weight during COVID-19 pandemic** (yes) | 219 (39.2) | 517 (41.6) | 0.561 |
| **Smoking during COVID-19 pandemic,** n: 556 | | | |
| More than before | 16 (10.3) | 121 (30.2) | **<0.001*** |
| Same as before | 34 (21.9) | 153 (38.2) | |
| Less than before | 11 (7.1) | 46 (11.5) | |
| I've started smoking | 2 (1.3) | 16 (4.0) | |
| I've quit smoking | 92 (59.4) | 65 (16.2) | |
| **Alcohol consumption during COVID-19 pandemic,** n: 1,085 | | | |
| More than before | 99 (36.3) | 98 (12.1) | **<0.001*** |
| Same as before | 137 (50.2) | 267 (32.9) | |
| Less than before | 36 (13.2) | 100 (12.3) | |
| I'm not drinking | 1 (0.4) | 347 (42.7) | |
| **Physical activity during COVID-19 pandemic,** n: 1,128 | | | |
| Yes | 216 (49.2) | 228 (33.1) | **<0.001*** |
| No | 91 (20.7) | 423 (61.4) | |

| | | | |
|---|---|---|---|
| No, but it was compensated by other exercise | 132 (30.1) | 38 (5.5) | |
| **Outdoors contact during COVID-19 pandemic** | | | |
| **Visits to natural environment** (yes), n: 1,429 | 288 (51.9) | 275 (31.5) | **<0.001*** |
| **Walking outside** (every day) | 214 (38.4) | 276 (22.2) | **<0.001*** |
| **Groceries shopping,** n: 591 | | | |
| Going as usual | 34 (22.1) | 175 (40.0) | **<0.001*** |
| Someone from my household | 44 (28.6) | 156 (35.7) | |
| Someone from outside my household | 11 (7.1) | 28 (6.4) | |
| Online or by phone | 65 (42.2) | 78 (17.8) | |

N=1800, unless otherwise specified. **Key;** OEC, other European countries; SD, standard deviation; UK, United Kingdom; * statistically significant at P<0.05.

**Table 2**. **Healthcare utilisation and psychological or psychiatry care during COVID-19 pandemic.**

| | **Mean ± SD; n (%)** | | **p-value** |
|---|---|---|---|
| | **UK** 558 (31.0%) | **OEC** 1,242 (69.0%) | |
| Healthcare utilisation | | | |
| Scheduled appointment with rheumatologist, n: 722 | | | |
| Yes | 87 (51.2) | 213 (38.6) | **0.004*** |
| Contact with rheumatologist about possible effects of treatment on COVID-19, n: 430 | | | |
| Yes | 94 (81.0) | 181 (57.6) | **<0.001*** |
| Access to primary care or general practitioner, n: 689 | | | |
| Yes | 87 (76.3) | 310 (53.9) | **<0.001*** |
| Psychological or psychiatry care | | | |
| Not continue psychological/psychiatry therapy (online or by phone), n: 437 | 16 (45.7) | 210 (52.2) | 0.459 |

N= 1,800, unless otherwise specified. **Key**; OEC, other European countries; SD, standard deviation; UK, United Kingdom; VAS, visual analogue scale; * statistically significant at P<0.05.

**Table 3. Disease-specific characteristics.**

| | Mean ± SD; n (%) | | p-value |
|---|---|---|---|
| | **UK** 558 (31.0%) | **OEC** 1,242 (69.0%) | |
| **Self-perceived health** | | | |
| Very good | 66 (11.8) | 59 (4.8) | **<0.001*** |
| Good | 236 (42.4) | 283 (23.0) | |
| Fair | 207 (37.2) | 595 (48.4) | |
| Bad | 47 (8.4) | 246 (20.0) | |
| Very bad | 1 (0.2) | 46 (3.7) | |
| **Change in health status during lockdown** | | | |
| Much worse than before | 23 (4.1) | 159 (12.9) | **<0.001*** |
| Moderately worse | 191 (34.3) | 459 (37.3) | |
| Same as before | 305 (54.8) | 538 (43.8) | |
| Moderately better | 34 (6.1) | 63 (5.1) | |
| Much better than before | 4 (0.7) | 10 (0.8) | |
| **VAS Disease activity** (≥4), n: 1,756 | 374 (67.6) | 920 (76.5) | **<0.001*** |
| **VAS Pain** (≥4), n: 1,421 | 373 (67.7) | 701 (80.6) | **<0.001*** |
| **WHO-5**, n: 1,777 | | | |
| Poor well-being (WHO-5≤50) | 292 (52.5) | 578 (47.3) | **0.043*** |
| **HADS anxiety (0-21),** n: 1,769 | | | |
| Risk (8-21) | 241 (43.6) | 772 (63.5) | **<0.001*** |
| **HADS depression (0-21)**, n: 1,769 | | | |
| Risk (8-21) | 186 (33.6) | 625 (51.4) | **<0.001*** |

N=1,786. **Key;** HADS, Hospital Anxiety and Depression Scale; OEC, other European countries; SD, standard deviation; UK, United Kingdom; VAS, visual analogue scale; WHO-5, the World Health Organisation five well-being index. * statistically significant at P<0.05.

**Figure 1. Information received about how COVID-19 might affect your rheumatic disease.** The information received about how COVID-19 might affect their condition was better rated by the UK sample (3.2 out of 5) versus the OEC (2.8 out of 5), and this difference was statistically significant ($p<0.001$). N=1682. **Key**; OEC, other European countries; UK, United Kingdom.

**Figure 2. Type of support from a Patient Organisation of patients. Key**; OEC, other European countries; UK, United Kingdom. N=331

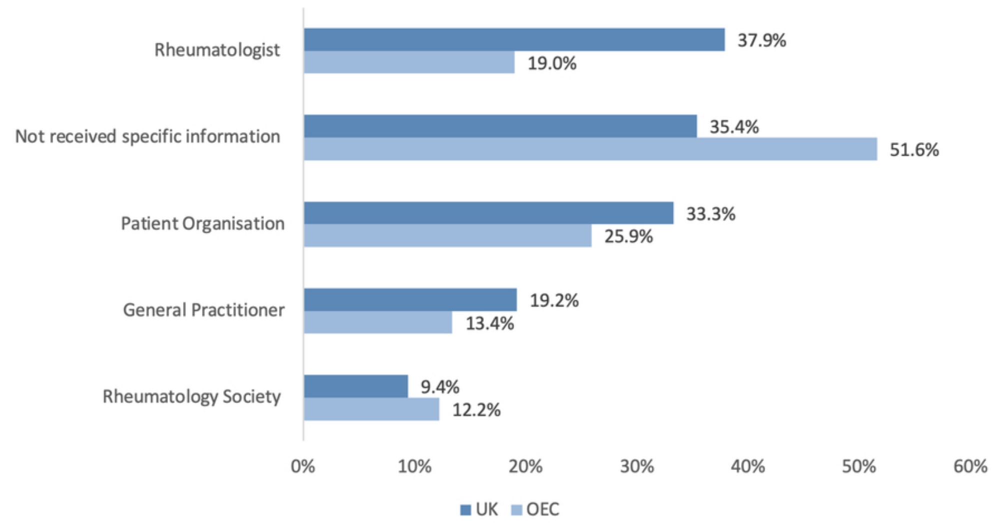

Figure 1. Information received about how COVID-19 might affect your rheumatic disease. The information received about how COVID-19 might affect their condition was better rated by the UK sample (3.2 out of 5) versus the OEC (2.8 out of 5), and this difference was statistically significant ($p<0.001$). N=1682. Key; OEC, other European countries; UK, United Kingdom.

75x41mm (300 x 300 DPI)

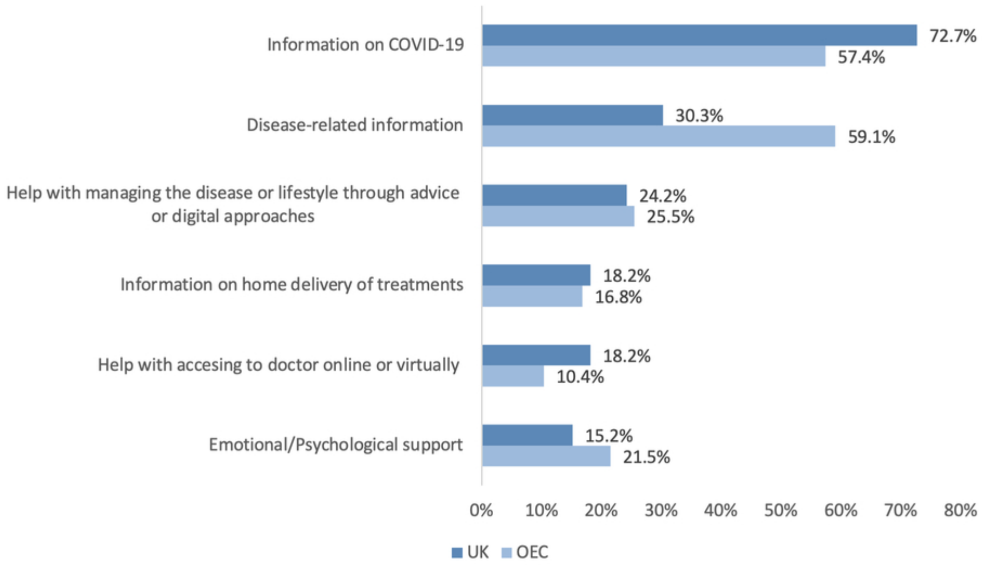

Figure 2. Type of support from a Patient Organisation of patients. Key; OEC, other European countries; UK, United Kingdom. N=331

78x46mm (300 x 300 DPI)
